# Supplementary material for: Structural basis of respiratory syncytial virus subtype-dependent neutralization by an antibody targeting the fusion glycoprotein
Source: Nat Commun. 2017 Nov 30;8:1877. doi: 10.1038/s41467-017-01858-w (PMC5707411; doi:10.1038/s41467-017-01858-w)
Supplement: Supplementary file 1 — Supplementary Information [file 41467_2017_1858_MOESM1_ESM.pdf]

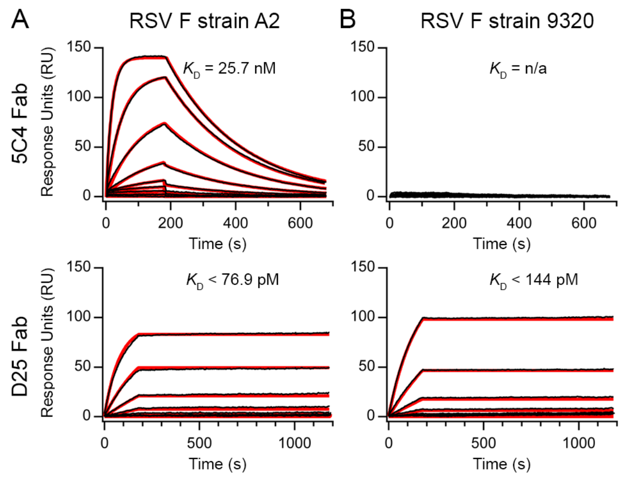

C

	strain A2		strain 9320	
	k_a ($M^{-1}s^{-1}$)	k_d (s^{-1})	k_a ($M^{-1}s^{-1}$)	k_d (s^{-1})
5C4	1.77×10^5	4.54×10^{-3}	n/a	n/a
D25	1.30×10^5	$< 1 \times 10^{-5}$	6.93×10^4	$< 1 \times 10^{-5}$

Supplementary Figure 1 | Surface plasmon resonance experiments. (A) Sensorgrams for binding of Fab 5C4 (*upper*) and D25 (*lower*) to prefusion-stabilized RSV F derived from strain A2. (**B**) Same as (A), except RSV F derived from strain 9320. For all panels, equilibrium dissociation constants (K_D) are listed. Red lines represent best fit of the data to a 1:1 binding model. (**C**) Kinetic rate constants derived from the best fits of the model.

A

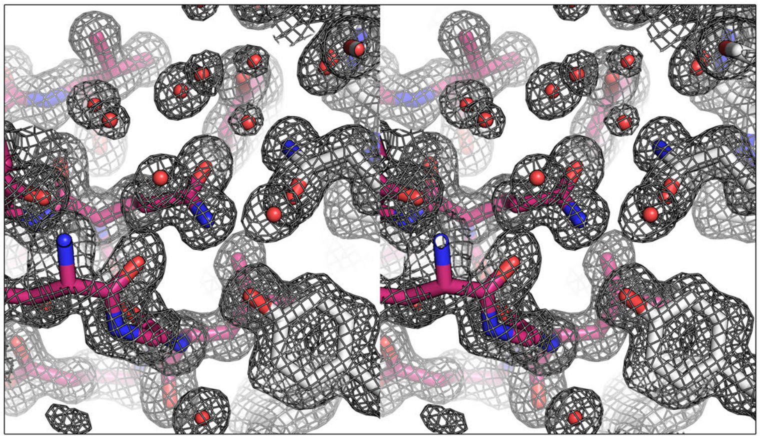

B

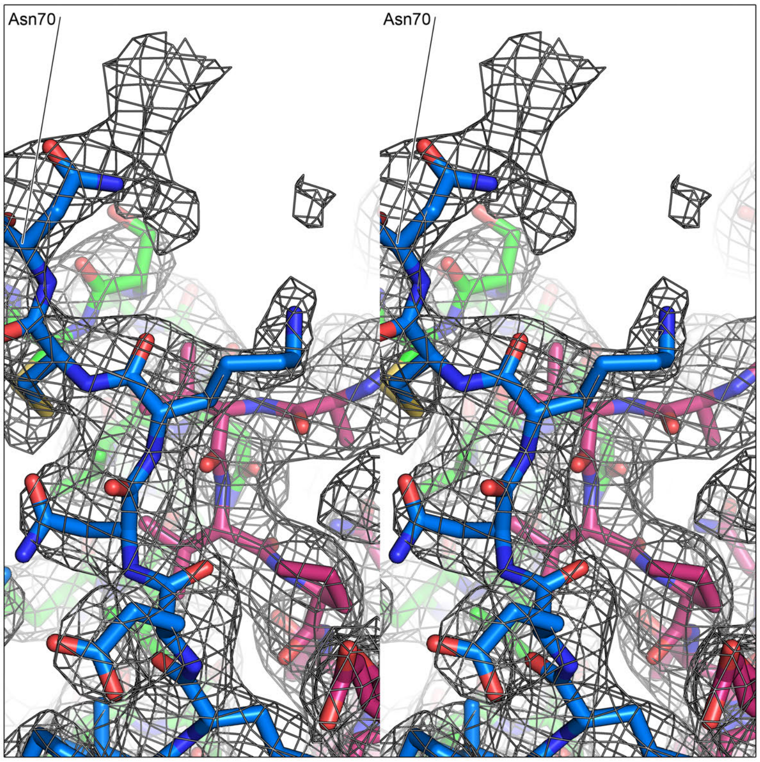

Supplementary Figure 2 | Stereo images of electron density. Stereo images of a portion of **(A)** the 5C4 Fab crystal structure and **(B)** the 5C4–RSV F complex crystal structure depicted as in Figure 2 with the $2F$o-Fc electron density maps contoured at 1σ shown as a black mesh.

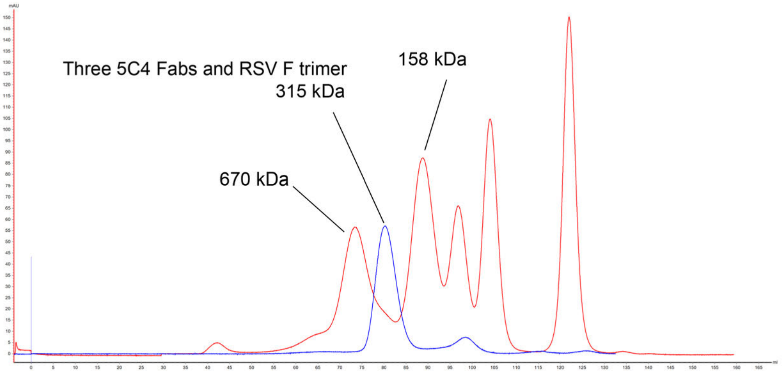

Supplementary Figure 3 | 5C4–RSV F complex has an approximate molecular weight of 315 kDa, consistent with a trimeric RSV F bound to three Fabs. 5C4–RSV F complex is shown as a blue trace and molecular weight standards (BioRad) are overlaid as a red trace with 670 kDa (bovine thyroglobulin) and 158 kDa (bovine γ-globulin) labeled for comparison.

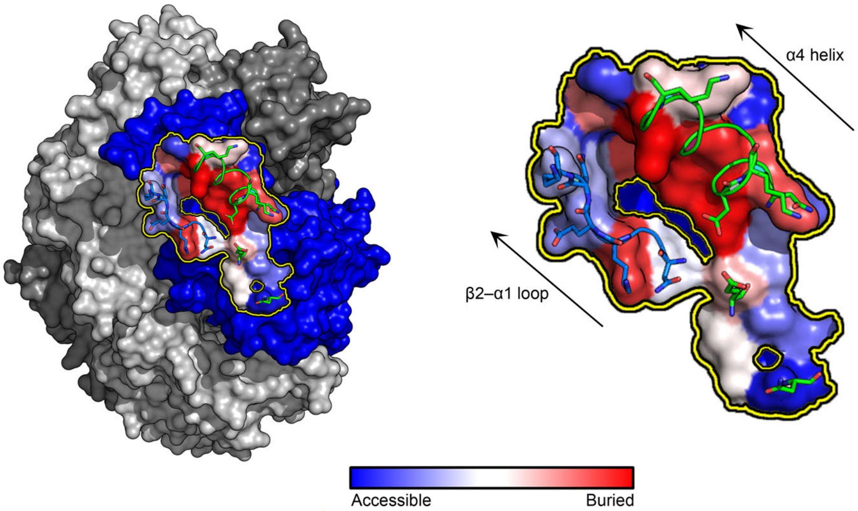

Supplementary Figure 4 | Surface of RSV F buried by 5C4. (*Left*) Two RSV F protomers are shown in gray and the remaining RSV F protomer is colored to show buried surface area (BSA). BSA is shown from blue to white to red (accessible to buried), calculated as the fraction of accessible surface area (ASA) on the RSV F protomer buried by 5C4 (BSA/ASA). The α4 helix of RSV F is colored green and the loop between β2 and α1 is colored blue, with select side chains shown as sticks. (*Right*) enlargement of the 5C4 binding footprint depicted as above.

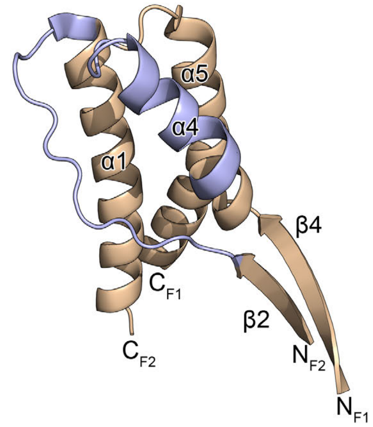

F1:	200	201	202	203	204	205	206	207	208	209	210	211	212	213
A2:	D	K	Q	L	L	P	I	V	N	K	Q	S	C	S
A(Long):	D	K	Q	L	L	P	I	V	N	K	Q	S	C	R
B18537:	N	N	R	L	L	P	I	V	N	Q	Q	S	C	R
B9320:	N	N	Q	L	L	P	I	L	N	Q	Q	S	C	R

F2:	60	61	62	63	64	65	66	67	68	69	70	71	72	73	74
A2:	E	L	S	N	I	K	E	N	K	C	N	G	T	D	A
A(Long):	E	L	S	N	I	K	E	N	K	C	N	G	T	D	A
B18537:	E	L	S	N	I	K	E	T	K	C	N	G	T	D	T
B9320:	E	L	S	N	I	K	E	T	K	C	N	G	T	D	T

Supplementary Figure 5 | Five subtype-specific mutations within site Ø. (*Left*) Site Ø epitope highlighted in light blue on a portion of a pre-F protomer colored wheat with annotated secondary structure. Carboxyl (C) and amino (N) termini are also labeled. (*Right*) Sequence of site Ø among select RSV strains. Residues that vary between subtype A and B are shaded in blue. Positions containing strain-specific variations are shaded in red.

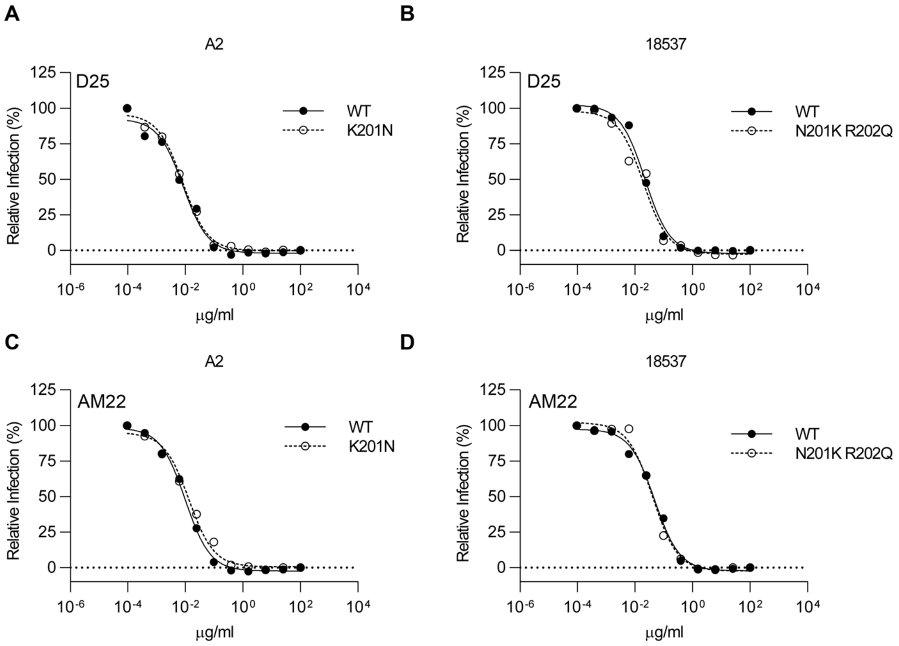

Supplemental Figure 6 | D25 and AM22 neutralize wild-type and position 201 mutant viruses of both subtypes with similar potency. (A) The EC_{50} of D25 against A2 wild-type and K201N mutant virus is 0.010 +/- 0.002 µg/ml (Mean+/-SD) and 0.008 +/- 0.0008 µg/ml, respectively. Data is representative of 3 independent experiments. **(B)** The EC_{50} of D25 against 18537 wild-type and N201K mutant virus is 0.029 +/- 0.008 µg/ml and 0.023 +/- 0.005 µg/ml, respectively. Data is representative of 3 independent experiments. **(C)** The EC_{50} of AM22 against A2 wild-type and K201N mutant virus is 0.015 +/- 0.005 µg/ml and 0.016 +/- 0.003 µg/ml, respectively. Data is representative of 3 independent experiments. **(D)** The EC_{50} of AM22 against 18537 wild-type and N201K mutant virus is 0.054 +/- 0.005 µg/ml and 0.046 +/- 0.003 µg/ml, respectively. Data are representative of three independent experiments.

Supplementary Table 1 Crystallographic data collection and refinement statistics

	5C4 Fab	RSV F with 5C4 Fab
**PDB ID**	5W24	5W23
**Data Collection**		
Space group	*C*2	$P4_12_12$
Cell dimensions		
a, b, c (Å)	117.0, 63.0, 56.5	183.4, 183.4, 275.9
α, β, γ (°)	90, 98.6, 90	90, 90, 90
Wavelength (Å)	1.000	0.979
Resolution (Å)	37.5–1.5 (1.53–1.50)*	34.1–3.4 (3.48–3.40)
R_{merge}	0.044 (0.234)	0.380 (1.596)
R_{meas}	0.051 (0.275)	0.406 (1.707)
R_{pim}	0.026 (0.143)	0.140 (0.585)
$I / \sigma I$	14.9 (3.8)	7.4 (1.9)
CC(1/2)	0.998 (0.938)	0.968 (0.462)
Completeness (%)	93.7 (69.7)	99.1 (99.2)
Redundancy	3.7 (3.5)	7.3 (7.2)
**Refinement**		
Resolution (Å)	32.9–1.5 (1.52–1.50)	34.1–3.4 (3.45–3.40)
Unique reflections	60,747 (2,024)	64,427 (2,773)
R_{work} / R_{free} (%)	17.0/20.3	19.8/23.9
No. atoms		
Protein	3,313	20,304
Solvent	6	8
Water	608	0
B-factors		
Protein	21.1	63.3
Solvent	25.3	106.0
Water	32.2	-
R.m.s. deviations		
Bond lengths (Å)	0.006	0.007
Bond angles (°)	0.85	1.05
Ramachandran		
Favored (%)	97.9	96.8
Allowed (%)	2.1	3.1
Outliers (%)	0	0
Clashscore	2.3	6.5

*Values in parentheses are for highest-resolution shell.

Supplementary Data 1. RSV F DS-Cav1 nucleotide sequences.

RSV A2_F DS-Cav1, codon optimized

ATGGAACTGCTGATCCTGAAGGCCAACGCAATTACCACTATCCTGACTGCCGTGACT
TTTTGTTTCGCAAGCGGACAGAACATTACAGAGGAGTTCTACCAGTCAACATGCAGC
GCCGTGTCCAAGGGATACCTGTCCGCTCTGCGAACCGGGTGGTATACATCTGTGATT
ACTATCGAGCTGAGTAACATCAAGGAAAACAAATGTAATGGCACCGACGCAAAGGT
GAAACTGATCAAGCAGGAGCTGGATAAGTACAAAAATGCCGTGACAGAACTGCAGC
TGCTGATGCAGAGTACACCAGCAACTAACAATCGCGCCAGGAGAGAGCTGCCCCGA
TTCATGAACTATACCCTGAACAATGCTAAGAAAACCAATGTGACACTGTCTAAGAA
ACGCAAGCGGCGCTTCCTGGGGTTTCTGCTGGGCGTGGGATCTGCCATCGCTAGTGG
GGTGGCCGTCTGCAAAGTCCTGCACCTGGAGGGCGAAGTGAACAAGATCAAATCAG
CTCTGCTGAGCACTAACAAGGCAGTGGTCAGTCTGTCAAATGGAGTGAGCGTCCTGA
CCTTTAAGGTGCTGGACCTGAAAAATTATATTGATAAGCAGCTGCTGCCTATCCTGA
ACAAACAGAGCTGTTCCATTTCTAATATCGAGACAGTGATCGAGTTCCAGCAGAAG
AACAATAGACTGCTGGAGATTACTCGGGAGTTCAGCGTGAACGCTGGCGTCACCAC
ACCCGTGTCAACCTACATGCTGACAAATTCCGAGCTGCTGTCTCTGATTAACGACAT
GCCTATCACCAATGATCAGAAGAAACTGATGTCTAACAATGTGCAGATCGTCAGAC
AGCAGAGTTATTCAATTATGTGCATCATTAAGGAGGAGGTCCTGGCCTACGTGGTCC
AGCTGCCACTGTATGGAGTGATCGACACCCCCTGCTGGAAACTGCATACAAGCCCTC
TGTGCACTACCAACACAAAGGAGGGGTCCAATATCTGCCTGACTCGAACCGACAGG
GGGTGGTACTGTGATAACGCAGGCAGCGTGTCCTTCTTTCCCCAGGCCGAGACCTGC
AAGGTCCAGAGCAACCGGGTGTTCTGTGACACTATGAATAGTCTGACCCTGCCTTCA
GAAGTCAACCTGTGCAATGTGGACATCTTTAACCCAAAGTACGATTGTAAGATCATG
ACTTCCAAGACCGATGTCAGCTCCTCTGTGATTACTAGCCTGGGAGCCATCGTGTCC
TGCTACGGGAAGACAAAGTGTACTGCTTCTAACAAAAACCGCGGCATCATTAAGAC
CTTTTCAAACGGATGCGACTATGTCAGCAACAAGGGCGTGGATACAGTGTCAGTCG
GAAACACTCTGTACTATGTCAATAAGCAGGAGGGGAAAAGCCTGTACGTGAAGGGC
GAACCAATCATTAACTTCTATGACCCCCTGGTCTTCCCTAGCGACGAGTTTGATGCA
TCTATTAGTCAGGTGAACGAAAAAATCAATCAGTCCCTGGCCTTTATTAGGAAGTCT
GATGAGCTGCTGCACAACGTGAATGCTGGCAAATCCACAACTAACATCATGATCAC
CACAATCATCATCGTGATTATCGTCATTCTGCTGTCTCTGATCGCTGTGGGACTGCTG
CTGTACTGTAAGGCAAGAAGTACCCCCGTGACCCTGAGCAAAGACCAGCTGAGCGG
CATCAATAACATCGCCTTCTCTAACTAATGA

RSV 18537_F DS-Cav1, codon optimized

ATGGAACTGCTGATTCATCGGAGTAGCGCAATTTTCCTGACACTGGCCGTGAACGCA
CTGTACCTGACCTCATCCCAGAACATTACCGAGGAATTCTACCAGAGCACATGCTCC
GCAGTGTCTAGGGGATACTTTAGCGCCCTGAGAACTGGGTGGTATACCAGCGTGATT
ACAATCGAGCTGTCCAATATCAAGGAAACCAAATGTAACGGAACAGACACTAAGGT
GAAACTGATCAAGCAGGAGCTGGATAAGTATAAAAATGCCGTCACAGAACTGCAGC
TGCTGATGCAGAACACTCCAGCCGCTAACAATCGAGCTAGGAGAGAGGCACCCCAG
TACATGAACTATACCATCAATACCACAAAGAACCTGAATGTGAGCATCTCCAAGAA
ACGCAAACGGCGCTTCCTGGGGTTTCTGCTGGGCGTGGGAAGCGCAATTGCCTCCGG
GATCGCTGTGTGCAAGGTCCTGCACCTGGAGGGCGAAGTGAACAAGATCAAAAATG
CCCTGCTGTCCACCAACAAGGCTGTGGTCTCTCTGAGTAACGGCGTGTCTGTCCTGA
CATTCAAGGTGCTGGACCTGAAAAACTACATTAACAATCGGCTGCTGCCTATCCTGA
ATCAGCAGTCTTGTCGCATTAGTAACATCGAGACTGTGATCGAATTCCAGCAGATGA
ATAGTCGCCTGCTGGAGATCACCCGAGAATTTTCAGTGAACGCCGGCGTCACTACCC
CTCTGAGCACTTACATGCTGACCAATTCAGAGCTGCTGAGCCTGATTAACGACATGC
CAATCACCAATGATCAGAAGAAACTGATGAGCTCCAACGTGCAGATCGTCAGGCAG
CAGTCATATAGCATTATGTGCATCATTAAGGAGGAAGTGCTGGCTTACGTGGTCCAG
CTGCCAATCTACGGCGTCATCGACACTCCCTGCTGGAAGCTGCATACCAGTCCTCTG
TGTACAACTAACATCAAGGAGGGAAGCAATATCTGCCTGACCAGAACAGACCGGGG
GTGGTACTGTGATAACGCTGGCTCCGTGTCTTTCTTTCCCCAGGCAGACACATGCAA
GGTGCAGTCCAATAGAGTCTTCTGTGATACAATGAACTCTCTGACTCTGCCTAGTGA
AGTGTCACTGTGCAACACAGACATCTTTAATTCCAAGTACGATTGTAAGATCATGAC
TTCTAAGACCGATATTTCTAGTTCAGTGATCACTAGTCTGGGAGCAATCGTCTCATG
CTACGGGAAGACAAAATGTACTGCCTCCAACAAGAATCGGGGCATCATCAAGACCT
TTTCTAATGGATGCGACTATGTCAGTAACAAGGGCGTCGATACCGTGTCTGTCGGAA
ACACACTGTACTATGTGAATAAGCTGGAGGGGAAAAACCTGTACGTCAAGGGCGAA
CCAATCATTAACTACTATGACCCCCTGGTGTTCCCTAGCGACGAGTTTGATGCTAGT
ATTTCACAGGTCAACGAAAAGATCAATCAGTCACTGGCATTCATTCGAAGGAGCGA
TGAGCTGCTGCACAACGTGAATACCGGCAAAAGCACCACAAACATCATGATCACTA
CCATCATCATCGTGATTATCGTGGTCCTGCTGAGCCTGATTGCCATCGGACTGCTGCT
GTATTGTAAGGCTAAAAACACCCCCGTGACACTGTCCAAGGATCAGCTGTCTGGGAT
TAACAATATCGCCTTTTCCAAATGATGA
